# Complete Genome Sequence of the Nonpathogenic Soil-Dwelling Bacterium *Clostridium sporogenes* Strain NCIMB 10696

**DOI:** 10.1128/genomeA.00942-15

**Published:** 2015-08-20

**Authors:** Aleksandra M. Kubiak, Anja Poehlein, Patrick Budd, Sarah A. Kuehne, Klaus Winzer, Jan Theys, Philip Lambin, Rolf Daniel, Nigel P. Minton

**Affiliations:** a The Clostridia Research Group, BBSRC/EPSRC Synthetic Biology Research Centre, School of Life Sciences, Centre for Biomolecular Sciences, University of Nottingham, Nottingham, United Kingdom; b Georg-August-University Goettingen Institute of Microbiology and Genetics, Department of Genomic and Applied Microbiology, Goettingen Genomics Laboratory, Göttingen, Germany; c Maastro Lab, Maastricht Radiation Oncology, University of Maastricht, Maastricht, The Netherlands

## Abstract

*Clostridium sporogenes* is a harmless spore-forming anaerobe that is widely distributed in soil/water and in the intestines of humans and animals. It is extensively used as a safe model to test the suitability of new preservative methods by the food industry and has potential to deliver therapeutic agents to tumors.

## GENOME ANNOUNCEMENT

*Clostridium sporogenes* NCIMB 10696 has the potential to be a tumor delivery vehicle for therapeutic agents (1, 2). It is classified by the UK Advisory Committee on Dangerous Pathogens as a harmless group I organism and by the American Type Culture Collection (ATCC 3584) as a harmless biosafety level 1 organism. Its categorization as safe is further demonstrated by the routine use of its spores by the food industry to assess the effectiveness of food preservation methods (3). While closely related to *Clostridium botulinum, C. sporogenes* does not produce botulinum toxin and differs markedly with regard to sporulation kinetics (4).

Genomic DNA was prepared by phenol-chloroform extraction (5) and sequenced using the 454 Titanium FLX (Roche Diagnostics) with 3 separate paired-end read sequencing libraries. Genomic DNA was also Illumina resequenced by GATC Biotech (Germany). The sequencing data (1,983,599 reads) were assembled using the GS assembler (Newbler 2.3) into 54 contigs (>500 bp) with a total size of 4,080,182 bp providing 74× coverage. The average contig length was 75,558 bp and the average scaffold size of 682,834 bp, with the largest scaffold being 3,498,261 bp. A total of 39 contig gaps within scaffolds were closed using standard PCR procedures, leading to a draft genome of 4,087,164 bp composed of 5 large scaffolds. Scaffold order was predicted using the move contigs tool of the Mauve Genome Alignment Software (6) and via Gene Ortholog Neighborhoods based on bidirectional best hits implemented at the IMG-ER (Integrated Microbial Genomes/Expert Review) system (7). Alignments were performed using the genomes of *C. sporogenes* ATCC 15579 (GenBank accession number ABKW00000000), *C. botulinum* ATCC 3502, and *C. botulinum* BoNT/B1 Okra (accession number CP000939) as references. A repeat of the assembly was carried out using the Newbler and Mira v3.4 packages and additional Sanger dideoxy sequencing. The final genome sequence length is 4,141,984 bp with an average coverage of 115×.

Gene prediction was performed using the software tool Prodigal (8). Genes encoding rRNA and tRNA were identified using RNAmmer (9) and tRNAscan (10), respectively. The IMG/ER (Integrated Microbial Genomes/Expert Review) system (7) was used for automatic annotation. Subsequent manual curation used the Swiss-Prot, TREMBL, and InterPro databases (11). The number of annotated features are coding sequence (CDS), 3,732; protein-encoding genes with function prediction, 2,956; putative genes coding for hypothetical proteins, 776; pseudogenes, 4; rRNA clusters, 9 (27 genes); and tRNA, 80.

The G+C content was 27.98%, and 82% of the genome was encoding. BLAST comparison revealed a 97 to 100% match to other *C. sporogenes* species and 91 to 99% to proteolytic *C. botulinum* strains (12, 13). The 16S rRNA gene sequences of *C. botulinum* and *C. sporogenes* strains share 99 to 100% nucleotide similarity. The assembly and annotation did not reveal the presence of any induced prophages or plasmids. BLAST and PCR analysis of the major toxins produced by *C. botulinum* (groups I through IV) demonstrated that no toxin-encoding genes or remnants are present, highlighting the nonpathogenic nature of *C. sporogenes* NCIMB 10696 and supporting its use in clostridial-directed enzyme prodrug therapy (CDEPT) (14, 15).

### Nucleotide sequence accession number.

The genome sequence is deposited at GenBank under the accession number CP009225.
